# Opinions and perceptions of patients with cardiovascular disease on adherence: a qualitative study of focus groups

**DOI:** 10.1186/s12875-024-02286-8

**Published:** 2024-02-16

**Authors:** Álvaro Carbonell-Soliva, Rauf Nouni-García, Adriana López-Pineda, Alberto Cordero-Fort, Virtudes Pérez-Jover, Jose A. Quesada, Domingo Orozco-Beltrán, Andreu Nolasco, Jose Maria Castellano-Vázquez, Jose Joaquín Mira-Solves, Vicente F. Gil-Guillen, Concepción Carratala-Munuera

**Affiliations:** 1 Network for Research on Chronicity, Primary Care. and Health Promotion (RICAPPS), Barcelona, 08007 Spain; 2 Clinical Medicine Department, School of Medicine, University Miguel Hernández de Elche, Alicante, 03550 Spain; 3 University Hospital of San Juan de Alicante, San Juan de Alicante, Alicante, 03550 Spain; 4 Institute of Health and Biomedical research of Alicante, Alicante Spain General University Hospital of Alicante, Diagnostic center, Fifth floor. Pintor Baeza street, 12, Alicante, 03110 Spain; 5 Foundation for the Promotion of Health and Biomedical Research of the Valencian, Community. N-332, 03550 San Juan de Alicante, Alicante, Spain; 6 Health Psychology Department, Miguel Hernandez University of Elche, Elche, Spain; 7 Cardiology Department, Hospital Universitario de San Juan, Alicante, Spain; 8 Biomedical Network Research Center for Cardiovascular Diseases (CIBERCV), Madrid, 28029 Spain; 9 Department of Community Nursing, Preventive Medicine, Public Health and History of Science, Faculty of Health Sciences, University of Alicante, San Vicente del Raspeig, Alicante, Spain; 10 Comprehensive Center for Cardiovascular Diseases, Montepríncipe University Hospital, HM Hospitales Group, Madrid, Spain; 11 General University Hospital of Elda, Alicante, 03600 Spain

**Keywords:** Perceptions, Adherence, Ischemic heart disease, Focus Group, semi-structured interview, Qualitative methodology

## Abstract

**Background:**

Cardiovascular diseases are becoming more frequent throughout the world. Adherence to both pharmacological and non-pharmacological treatment, as well as lifestyles, is important for good management and control of the disease. This study aims to explore the opinions and perceptions of patients with ischemic heart disease on the difficulties associated with therapeutic adherence.

**Methods:**

An interpretive phenomenological study was carried out using focus groups and one semi-structured interview. The MAXQDA qualitative data analysis program was used for inductive interpretation of the group discourses and interview. Data were coded, and these were grouped by categories and then consolidated under the main themes identified.

**Results:**

Two in-person focus groups and one remote semi-structured interview were performed. Twelve participants (6 men and 6 women) from the Hospital de San Juan de Alicante participated, two of them being family companions . The main themes identified were aspects related to the individual, heart disease, drug treatment, and the perception of the health care system.

**Conclusions:**

Adhering to recommendations on healthy behaviors and taking prescribed medications for cardiovascular disease was important for most participants. However, they sometimes found polypharmacy difficult to manage, especially when they did not perceive the symptoms of their disease. Participants related the concept of fear to therapeutic adherence, believing that the latter increased with the former. The relationship with health professionals was described as optimal, but, nevertheless, the coordination of the health care system was seen as limited.

**Supplementary Information:**

The online version contains supplementary material available at 10.1186/s12875-024-02286-8.

## Background

 Cardiovascular diseases (CVD) are chronic diseases that evolve gradually throughout a person’s lifetime, remaining asymptomatic for an extended period of time [1]. They are caused by multiple factors, some of which are not modifiable, such as age, sex, and genetics. On the other hand, others are amenable to preventive strategies and behavioral modifications that can influence the appearance and the course of the disease. These factors include tobacco use, physical inactivity, poor diet, high blood pressure, type 2 diabetes mellitus, dyslipidemia, and obesity [2].

Previous studies have shown that most recurrent cardiovascular events occur in survivors of an acute coronary syndrome, especially during the first year following the event [3]. Poor therapeutic adherence to treatment is related to worse health outcomes, and this in turn is generally more prevalent in patients with chronic health problems [4].

The World Health Organization (WHO) defines therapeutic adherence as “the extent to which a person’s behaviour – taking medication, following a diet, and/or executing lifestyle changes, corresponds with agreed recommendations from a health care provider” [5]. In this line, the following alternative definition, specifically related to medication adherence, is also accepted: “the process by which patients take their medication as prescribed, further divided into three quantifiable phases: ‘Initiation’, ‘Implementation’ and ‘Discontinuation’” [6].

Therapeutic adherence is conditioned by five interrelated factors [7], related to: the patient (e.g., age, employment or economic situation, culture, educational level, geographical area) [8–10], the disease, the therapeutic regimen, the health system, and the health professional [11, 12].

Numerous theories have been applied to explain patient behavior around therapeutic adherence [13, 14]. The information-motivation-behavioral skills model stands out in this regard [14], representing a model of social behavior in patients with chronic diseases in which motivation plays an important role. According to this model, the following three dimensions directly influence adherence behavior: (1) information and knowledge about the need for the essential behavior, (2) motivation to make the necessary behavior changes, and (3) the behavioral skills required to achieve the desired behavior.

Patient motivation is crucial when coping with multiple drug prescriptions and incorporating them into one’s daily routine [15]. Likewise, the literature describes several specific barriers to adherence, related to both the organization itself (external factors) [16] and to the individual (internal factors) [17], which can lead to undesirable behaviors regarding taking medication.

In Spain, the adherence rate in secondary prevention is 56% in patients with a previous cardiovascular event [18, 19]. In the European Union, the number of deaths associated with non-adherence to prescribed medication is 194,500 people per year, with an estimated cost of EUR 125 billion [20].

The magnitude of the problem, in terms of the low adherence in this patient population as well as the high number of deaths associated with it, warrants a deep dive into the difficulties perceived by patients themselves in order to understand the reasons behind the poor adherence rates. Thus, this study aims to explore the opinions and perceptions of people with ischemic heart disease regarding the difficulties associated with adherence to treatment.

## Materials and methods

### Study design

This interpretive phenomenological study, framed within a qualitative social research approach, was based on in-person focus groups. In line with the principles proposed by Lincoln and Guba [21], the criteria for trustworthiness applied were credibility, transferability, and confirmability. A focus group is a carefully planned conversation, designed to obtain information from a defined area of interest in a non-directive environment and to generate discourses among the participants. The discussion should be relaxed, comfortable and satisfactory for the participants, since they present their ideas and comments in a group of people with whom they have certain experiences in common; qualitative data are obtained in a guided and recorded conversation for later reproduction [22]. The COREQ checklist (Consolidated criteria for Reporting Qualitative research) [23] was used to carry out this study, which was approved by the Responsible Research Office of Miguel Hernández University (ref: DPC.RNG.01.22).

### Sampling method

The study participants were selected by purposive sampling from the patients attending the cardiology clinic of the San Juan University Hospital (Alicante, Spain) from March to June2022. This type of sampling allows for the intentional inclusion of individuals who fit the desired study profile. It is designed to select participants who ensure the optimal quantity and quality of information [24].Thus, Homogeneous and heterogeneous variables (common and differentiated characteristics) were established to create diverse participant profiles and foster the generation of discourses from the focus groups. The homogeneous variables were minimum age of 18 years, belonging to the same health department, and presenting the same disease (ischemic heart disease). The heterogeneous variables, defined to attain a group with a variety of perspectives, were: gender (men/women), employment status (actively employed, unemployed, retired, homemaker, others), marital status (married, divorced, single, widowed), and province of residence). Before the start of the focus groups, the participants were given the information sheet and signed informed consent to participate in the study and have their group sessions recorded in audio or video format. They were informed that the data collected were not going to be used publicly or for any purpose other than research.

### Participant selection

Eligible participants had to be aged 18 years or older, have ischemic heart disease, be in treatment for at least one year, and sign informed consent. Exclusion criteria were: having a neurodegenerative disease or significant cognitive problems that prevented the construction of speech and interaction with the rest of the participants, and/or hearing or language-related problems that make verbal communication difficult. It will not be mandatory for accompanying family members of the patients to meet the inclusion criteria.

All in all, two focus groups were created. Six male patients were invited to participate in the first (focus group 1, FG1), and six female patients in the second (focus group 2, FG2). Patients were encouraged to invite their main living companion to the group discussions to share their perspectives around therapeutic adherence. After having a routine consultation with their attending cardiologist and accepting the invitation to participate in the study, the patients were contacted by telephone and informed of the date and place of the focus group.

The final sample was made up of six women (4 patients and 2 living companions of the male patients in the first focus group) and six men (all patients). The first focus group, held on 25 March 2022, included the six male patients and two female cohabitants (8 participants in total). In the second, held on 17 June 2022, only three female patients were able to attend; since most of them were primary caregivers, their availability was more limited.

One of those who could not attend the focus group but who was keen to participate agreed to give an individual interview by video call. Thus, a 30-min, semistructured individual interview (SII) was held with her on 10 June 2022. In this case, one researcher conducted the interview using a previously prepared script and another researcher observed: an Additional File shows this in more detail (Additional file 1).

### Focus group guide

The correct management of the focus groups was ensured through the use of a guide previously prepared by the research group, an Additional File shows this in more detail (Additional file 2), structured into three thematic blocks, with considerations to be taken into account prior to starting the focus group conversations, during their course, and following the end of the discussions. In addition, a list of flexible, open-ended questions was proposed as a reference and support for managing the discourse, in order to allow informants the time and space to express their own points of view without any type of prior conditioning [25, 26].

### Group dynamics

The setting chosen for the meeting was intentionally outside the health care center (conference room of the Miguel Hernández University Clinical Medicine Department) to favor a relaxed atmosphere in which the participants felt free to express themselves. The focus groups lasted approximately 60 min and were moderated by a health researcher with extensive experience in this type of qualitative methodology. Three researchers were present as observers, collecting relevant notes for the subsequent analysis of the information and managing the audio and video recording of the sessions. In FG1, participants’ companions signed a different informed consent form than the patients, without the items referring to cardiovascular disease and treatment and with a new item inquiring whether or not they were the main caregiver.

### Data analysis

Participants’ sociodemographic characteristics (sex, age, place of residence, employment situation, marital status) were collected, along with information on whether they were the main caregiver in their home, the years taking cardiovascular medication, and the time since the first infarction (in participants with ischemic heart disease). These variables were categorized, and frequencies and percentages were calculated.

The audio and video recordings of the focus groups were used to manually transcribe the data and emerging discourses in a Microsoft Word document. To obtain a thematic description of the data, three researchers undertook a first inductive analysis with open codes, based on the focus group discussions, and shared them with the research team 15 days later. Once a preliminary consensus was reached, two researchers performed inductive coding with open codes through the qualitative data analysis software program MAXQDA [27]. A deviant case analysis was carried out to identify data elements that were not consistent with the main themes [28]. The open coding process was carried out from the most general to the most specific aspects [29], generating an analysis scheme made up of codes, which were grouped into categories and then consolidated into main themes (Table 1).

**Table 1:** Process of discourse interpretation: codes, categories, and main themes

Codes	Categories	Main themes
­ Responsible for taking care of themselves ­ Doing exercise ­ Smoking ­ Diet ­ Having a normal life ­ Meeting with friends ­ Meals away from home	a. Lifestyle	1. Aspects related to the individual
­ Beliefs about medication ­ Beliefs about the disease	b. Personal beliefs	
­ Absence of fear ­ Presence of fear	c. Fear	2. Aspects related to heart disease
Presence of symptoms: ­ Fatigue ­ Bruising ­ Head pressure ­ Swelling ­ Chills ­ Cold sweats ­ Nausea ­ Weakness Absence of symptoms	d. Disease symptomology	
­ Importance of taking medication ­ Distrust of medication	e. Beliefs about medication	3. Aspects related to drug treatment
­ Taking pills every day ­ Lack of adherence to drug treatment ­ Forgetting to take pills sometimes ­ Polypharmacy ­ Medication side effects ­ Reminder systems for taking medication	f. Taking medication	
­ Lack of information ­ Lack of coordination ­ Trust ­ Accessibility ­ Communication with health professionals	g. Patient-health system and health professional relationship	4. Aspects related to the perception of the health care system

This content coding process was flexible and open to possible modifications, with the participation of an external expert in qualitative data analysis tools, who supervised the use of the analysis tool and the coding process. Group meetings were held with the rest of the research team, and discrepancies between observers were resolved through consensus.

**Fig. 1:**
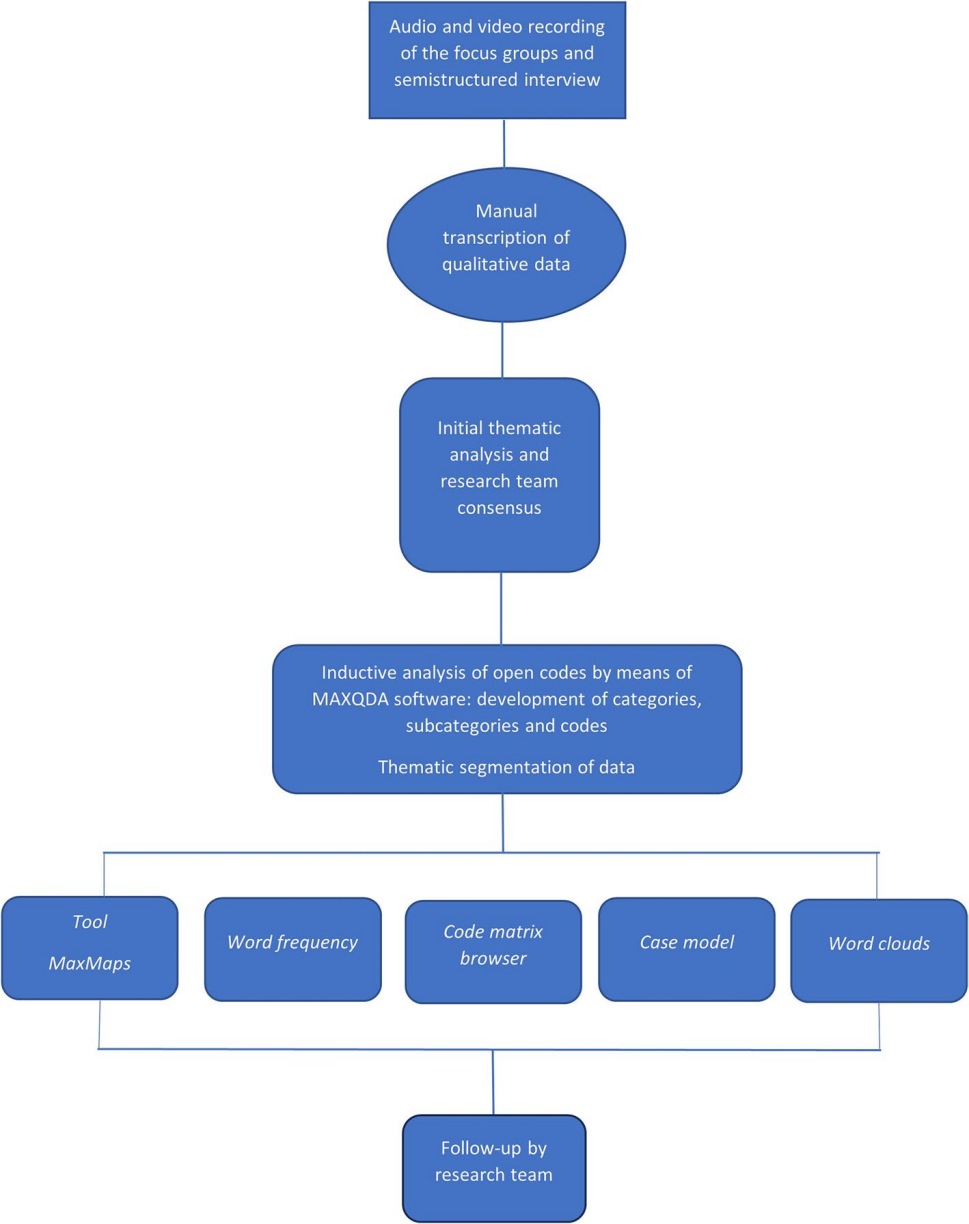
Flow chart for data analysis process

Following the line of inductive content analysis, MAXMaps tools such as word clouds, the code matrix browser, and the case model were used to identify the main categories (Fig. 1).

## Results

Table 2 shows the sociodemographic characteristics of all study participants.

**Table 2:** Sociodemographic characteristics of the sample of participating patients (*N* = 10) and living companions (*N* = 2)

Characteristics	N (%)
Gender	
Men	6 (50.0)
Women	6 (50.0)
Age group	
40–49 years	1 (8.3)
50–59 years	3 (25.0)
60–69 years	6 (50.0)
70–79 years	2 (16.7)
Province	
Alicante	12 (100.0)
Employment status	
Actively employed	3 (25.0)
Unemployed	2 (16.7)
Retired	6 (50.0)
Homemaker	1 (8.3)
Years taking drug treatments for cardiovascular disease	
1–5	2 (20.0)
6–10	3 (30.0)
11–15	3 (30.0)
+ 15	2(20.0)
Years since first heart attack	
1–5	2 (20.0)
6–10	3 (30.0)
11–15	3 (30.0)
+ 15	2 (20.0)
Marital status	
Married	9 (75.0)
Widowed	3 (25.0)
Primary caregiver	
Yes	2 (16.7)
No	10 (83.3)

**Fig. 2:**
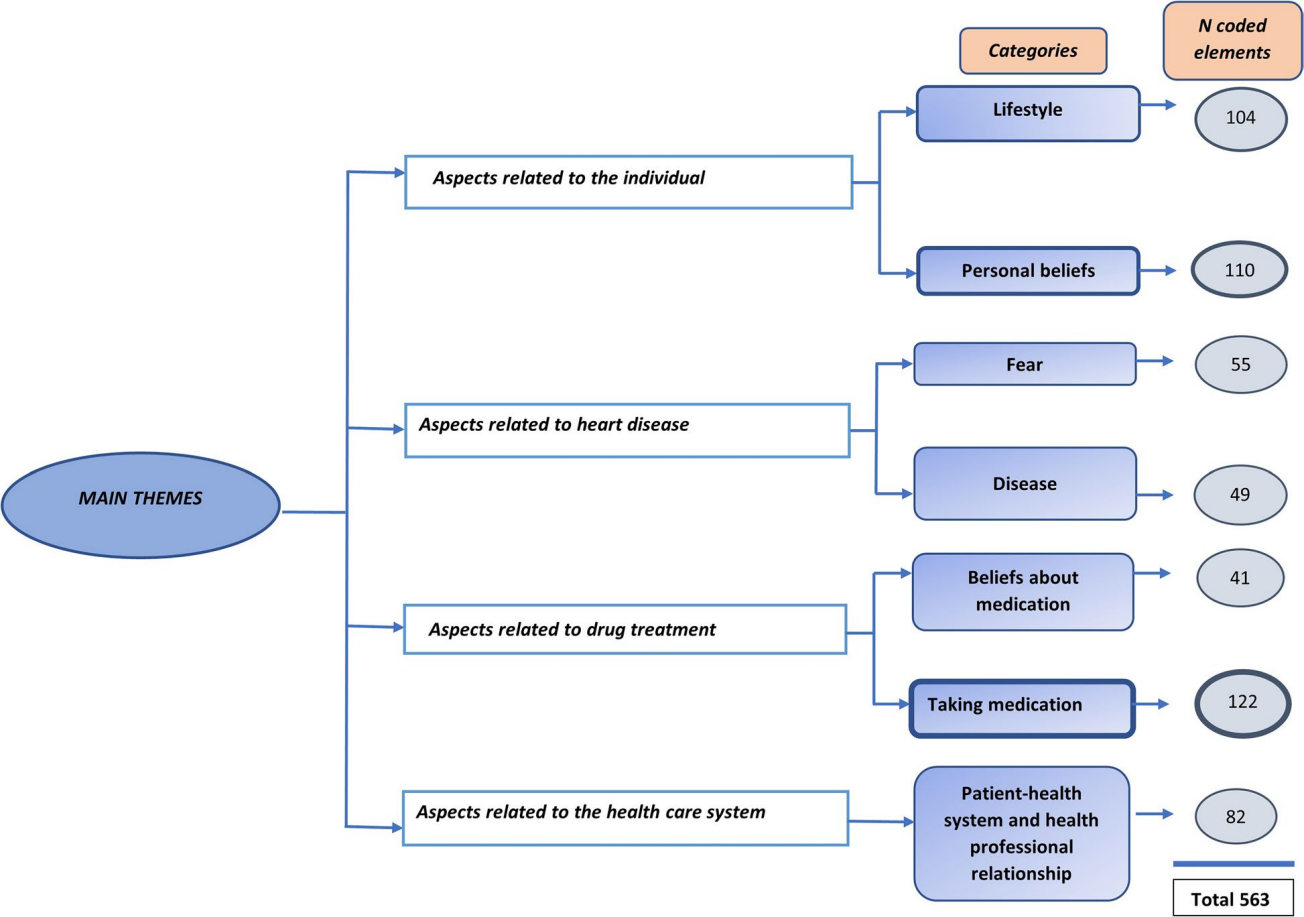
Coding scheme and number of coded elements per category. The thickness of the border indicates the volume of coded elements in that category

Altogether, the qualitative data yielded 563 codes (FG1, *n* = 336; FG2, *n* = 175; and SII, *n* = 52). Subsequently, the identified codes with a common meaning were consolidated and interpreted, with seven categories emerging. In a second phase, these categories were synthesized and grouped into four main themes, covering aspects related to the individual, heart disease, drug treatments, and the perception of the health care system (Fig. 2). Table 1 presents a description of the most frequent codes by category and main theme.

The topics most commonly reiterated by participants, that is, the categories with the highest number of coded data elements, were: taking medication (*n* = 122 codes), personal beliefs (*n* = 110), and lifestyle (*n* = 104) (Fig. 2). The study findings are detailed below according to the main themes and corresponding categories that emerged during the analysis.

### Aspects related to the individual

#### Category: lifestyle

Most study participants considered it important to take care of themselves, be responsible for managing their health, lead an active and healthy life, and be knowledgeable about the disease they had in order to guide their behavior and respect the associated limitations. While they generally recognized the importance of adhering to a healthy, low-fat diet and maintaining a good general state of health, several found it difficult, and sometimes even boring, to strictly follow such restrictive diets, and admitted to being unable to resist temptations with regard to unhealthy foods, especially on special occasions associated with leisure and gatherings of family or friends.

In the case of the focus group in female patients, tobacco was a prominent topic of the group discussion, as all had been heavy smokers for much of their lives. One had managed to quit a few years before, while the other two continued to smoke and described the impossibility of quitting after numerous failed attempts; they said that smoking brought them pleasure, and they were not considering quitting at that time. Furthermore, one of the FG2 participants admitted that she enjoyed eating, and that she was not considering improving her diet under any circumstances, even though sometimes she ate until feeling stuffed; on this point, her perspective differed from the rest of the participants in the focus group. Supplementary Table S1 shows the coded quotations and the participant codes.

#### Category: personal beliefs

Participants in both focus groups expressed their personal beliefs repeatedly throughout the group discussions, especially about two main topics: medication and CVD. Regarding the beliefs around medication, the predominant opinion was that it is essential to achieve and maintain good health, since taking care of oneself is frequently associated with taking medication. Only one FG2 participant differed from the rest of her group, expressing her open mistrust towards certain types of medications that she doubted really worked to improve her health. The SII participant reported that medication is something essential and important for her to maintain her quality of life, so she took it daily and described it as something good and positive in her life.

Regarding the beliefs about the disease, participants commonly expressed the opinion that a diagnosis should not lead to a limited life and highlighted the importance of leading a normal, active life, without giving up their previous activities. In this line, fear was a recurrent topic during FG1, with participants describing its huge impact on their lives, especially just after their diagnosis, which limited them in certain situations, although they progressively overcame it as they got used to living with the disease. Supplementary Table S2 shows the coded quotations and participant codes.

### Aspects related to heart disease

#### Category: fear

Regarding aspects related to heart disease, fear was the most reiterated concept among the FG1 participants, both its absence and its presence. On the one hand, FG1 participants considered fear to be understandable and normal, especially right after the diagnosis of the disease. After a certain period of time has passed, though, the fear subsides, and the resulting complacency commonly leads to the person to eventually fall back into unhealthy habits or neglect therapeutic adherence. On the other hand, participants described the presence of fear permeating numerous life situations, leading to attitudes of hypervigilance and excessive caution, even generating feelings of anxiety and sleep disturbances in some patients. One participant commented that one cannot live with fear long term, since the feelings end up being worse than the disease itself.

In FG2, the general attitude, especially in two of the participants, was one of resignation to the disease, with a feeling of accepting the occurrence of an event if it had to happen. In general, they preferred to continue unhealthy behaviors, such as smoking or eating what they wanted, if it made them happy or brought them pleasure. In this focus group, fear was mentioned more frequently in relation to the side effects caused by certain medications according to their own research on the internet. Supplementary Table S3 shows the coded quotations and participant codes.

#### Category: disease symptomology

Most participants in FG1 reported that the most frequent symptoms of CVD that they experienced were excessive sweating, chills, and fatigue. Other FG1 participants described joint pain, vomiting, and dizziness prior to diagnosis. They recounted that alarm signals that pushed them to go to the hospital urgently were sweating, nausea, dizziness, and chills, since they were accompanied by a feeling of continuous general malaise. However, after the diagnosis and the start of treatment, with the exception of tiredness and moments of fatigue, most reported that in their day-to-day life they did not perceive any notable symptoms with a negative impact on their health. In that line, one of the women accompanying a participant in FG1 mentioned it was exactly this absence of symptoms characteristic of the disease that led her partner to downplay it and to underestimate the importance of taking care of himself.

In FG2, the comments related to symptomology were less frequent than in FG1, with fatigue being the most commonly mentioned symptom. One participant shared that she had stopped taking one of the drugs for a few days to see the effects, and that she began to feel more fatigued. Likewise, she reported that she retains a lot of fluids and often feels bloated. On the other hand, another participant commented that she is sometimes tired, but she thinks it may be due to age. Finally, the woman who was interviewed said that after the diagnosis and the start of treatment, she had never felt any symptoms. Supplementary Table S4 shows the coded quotations and participant codes.

### Aspects related to drug treatment

#### Category: beliefs about medication

Regarding participants’ beliefs about medication for CVD, the perceptions of all the participating patients were generally positive. Most of the FG1 participants considered medication to be crucial, and important to take every day. Both a female companion from FG1 and a participant from FG2 said that, for them, “medication is sacred”. The participant interviewed remotely commented that she would be willing to take all the pills prescribed by the doctor, because for her maintaining a good quality of life was the most important thing, and she was facing the disease process with optimism and discipline.

This generally positive perception towards medications was not universal. One of the FG2 participants reiterated on numerous occasions that she still has doubts about the effectiveness of the pills she takes and whether they actually help her. She even commented that some of them could cause adverse effects and confirmed that she sometime deliberately stops taking some pills to check their effect on her health. Supplementary Table S5 shows the coded quotations and participant codes.

#### Category: taking medication

Generally, participants described their medication adherence as good, and most reported taking it routinely. Only one of the FG2 participants disagreed with the rest, admitting that on some occasions she intentionally stopped taking certain drugs. Participants also discussed occasionally forgetting to take the medication, mostly due to leisure activities outside the home with family or friends that break their routine, leading them to leave the medicines at home or forgetting to take them. In general, the participants downplayed the consequences that these occasional slipups could entail.

Regarding the medication reminder systems, such as pillboxes, bags with medications, and notes, these were widely used by the participants, most of whom recognized their usefulness for making sure to take their medication and systematize their organization.

Most participants presented polypharmacy, largely due to other comorbidities apart from CVD, such as diabetes. This situation was widely described as difficult to manage as well as exhausting. Supplementary Table S6 shows the coded quotations and participant codes.

### Aspects related to the perception of the health care system

#### Category: patient-health system and health professional relationship

In this category, both in the focus groups and in the interview, the perceived relationship with health professionals was positive. Most participants agreed that their follow-ups were individualized to their specific situation and that they got a good explanation after the diagnosis of the guidelines to follow regarding the medication and the function of each drug. However, the times between consultations are quite long (some reported intervals of a year between appointments), which makes continuity of care difficult. In addition, FG1 participants express a perceived deficiency in health system coordination, particularly among distinct Autonomous Communities, with respect to electronic medical records access and monitoring.

One of the FG2 participants, despite commenting that she has a positive view of health professionals in general, especially with regard to communication with her and with the treatment received by young professionals, openly admitted that she does not ask the doctors about issues related to her health, because she believes that they do not have time to respond to her particular queries. An FG1 participant commented that the hospital’s cardiac rehabilitation service following a heart attack is an investment in health, and he maintains a good perception of the multidisciplinary team of professionals who work in the previously mentioned service. The coded quotations and participants are shown in Supplementary Table S7.

## Discussion

Our findings suggest that personal beliefs regarding medication and illness have considerable weight in terms of the difficulties associated with medication adherence, highlighting that patients who believe in the importance of taking medication correctly and having healthy habits tend toward good therapeutic adherence. The challenge of maintaining a healthy lifestyle figured more prominently in the focus group made up of women than that one in men, with participants stating that changing unhealthy behaviors such as an unbalanced diet and smoking was a problem. On the other hand, fear was a more recurrent topic in the focus group of men, who associated this feeling with better therapeutic adherence. Other studies published in Spain by Isaza et al. [30] and Fernández-Arias et al. [31] agree that it is essential for professionals to promote positive attitudes and beliefs regarding medication in order to increase patients’ adherence to drug treatment. A study published in Mexico by Suárez-Arguello et al. [32] is consistent with our findings, concluding that better adherence is associated with having realistic views about the disease and positive beliefs about the benefits of the medication.

Regarding aspects related to heart disease, the absence of fear, associated with the accumulation of time living with the disease and the absence of characteristic symptoms, seemed to be related to the lack of therapeutic adherence. As for aspects related to drug treatment, participants generally had positive perceptions about the benefits of taking the medication, although there were a few mentions—especially from the female participants—about the side effects of the medication. Similarly, participants had generally positive opinions about the care received by health professionals, despite their agreement about the lack of coordination in the health system. These findings coincide with those observed in several previous studies, such as those by Vries McClintock et al. [33], Smeltzer et al. [34], and Ferguson et al. [35] regarding the lack of health provider coordination as a potential factor contributing to non-adherence.

Polypharmacy was a common characteristic among all the participants. In this line, other studies agree that polypharmacy and the complexity of the treatment represent a challenge in patients with cardiovascular problems [36–38], especially in patients who report that their disease is asymptomatic and who thus tend to underestimate its severity [36]. Therefore, our findings underscore the need to establish a stronger collaboration between family doctors and other health professionals to address the problems of polypharmacy in this population of patients.

In addition, a recent study in France, by Sidorkiewicz et al. [39], noted a discrepancy between patient and physician assessments of medication adherence and the importance of medication, which could be caused by a lack of patient-centered communication. In this context, there are studies that show the importance of patient-centered care in promoting therapeutic adherence and self-efficacy in medication management [40–44], with special attention paid to the importance of establishing communication between the professional and the patient to encourage adherence to treatment [45].

Among the barriers to therapeutic adherence identified in the literature, numerous studies have mentioned the feelings of fear and anxiety in patients due to the description of possible side effects and drug interactions commonly described in the package inserts [34, 46–50]. This concern was also brought up by one of the female participants in our study. This phenomenon can be interpreted as a potential contributor to non-adherence, intricately linked to individual beliefs regarding medications and their perceived adverse effects on health [51]. Emphasizing health education, as underscored in various prior studies, becomes crucial [52, 53]. This facilitates patient empowerment in disease management, enhancing their understanding of drug purposes and potential interactions.

Regarding the limitations of the study, one of the most frequent in qualitative studies based on focus groups is the possible lack of knowledge or opinion among some participants regarding the topic addressed. To overcome this limitation, in the participant selection phase, those who could best address the research objective were selected, that is, those who are familiar with the phenomenon of chronic illness and taking medication are selected.” (xx). Furthermore, due to the influence of traditional gender roles, it was challenging to achieve the participation of female participants, and we were unable to reach the expected number of women in FG2, since many women did not have enough time to take part after working in their job and caring for others at home. In an effort to minimize the impact of this limitation, we proposed a remote interview for one interested participant. These difficulties highlight a potentially generalized barrier to gender-inclusiveness in qualitative research on this topic. Future studies are needed that specifically tailor their design to ensure the inclusion of women who may have other obligations that compete with their participation in such research, as the difficulties they face in adhering to their therapeutic regimen could very well be different from those faced by people with more time availability.

Finally, in order to control the unintentional influence of the moderator on the group discourse, she adopted a neutral attitude throughout the course of the focus groups.

As for the strengths of the study, the sampling technique took into account the homogeneity and heterogeneity of the participants, since men and women with a common problem (therapeutic adherence in cardiovascular disease as criteria of homogeneity) but different profiles (age, sex, and family and socioeconomic variables as criteria of heterogeneity) were included to guarantee fluidity, variability, and richness in the discourses generated. Moreover, the support provided by the qualitative analysis program MAXQDA was useful in the process of filtering, coding, and categorizing the data, which, as in the vast majority of qualitative research, occurs in large quantities.

## Conclusions

In relation to the main themes, the conclusions obtained were as follows.

### Aspects related to the individual

The male participants stressed the importance of adopting healthy behaviors for good control of the disease, while the women, despite acknowledging the importance of a healthy lifestyle, were not willing to give up unhealthy habits such as tobacco or an unbalanced diet because it brought them happiness.

### Aspects related to heart disease

Participants’ personal beliefs revolved around the concepts of medication—considered essential for disease control, and cardiovascular disease—closely related to the concept of fear. The absence or presence of fear was seen as influencing medication adherence, with participants arguing that the greater the fear, the greater the motivation for taking the medication, while its absence leads to complacency in terms of behavior and medication adherence.

### Aspects related to drug treatment

Disease symptoms usually go unnoticed by the participants, which paradoxically increases the perceived burden of taking a large number of pills every day to control CVD.

### Aspects related to the perception of the health care system

The relationship with health professionals is optimal, but there are certain limitations in the coordination of the health system that sometimes make the care process difficult.

### Supplementary Information

**Additional file 1.**

**Additional file 2.**

**Additional file 3.**

## Data Availability

The data that support the findings of this study are available from the corresponding author, [ALP], upon reasonable request.

## Abbreviations

FG: Focus Group
SII: Semistructured Individual Interview
COREQ: Consolidated Criteria for Reporting Qualitative Research

## Declarations

The participants in this study signed an informed consent prior to inclusion in the study by which the personal willingness to participate was recorded in writing.
