# Supplementary material for: Opinions and perceptions of patients with cardiovascular disease on adherence: a qualitative study of focus groups
Source: BMC Prim Care. 2024 Feb 16;25:59. doi: 10.1186/s12875-024-02286-8 (PMC10870481; doi:10.1186/s12875-024-02286-8)
Supplement: Supplementary file 1 — Additional file 1. [file 12875_2024_2286_MOESM1_ESM.docx]

**Additional File 1**

***Script for the semistructured individual interview***

Date of interview: 10 June 2022

Modality: Zoom Meetings

**Things to keep in mind before the interview:**

First, we receive and greet the participant.

Subsequently, we thank her for her participation and introduce the researchers. Then we invite the participant to introduce herself.

We proceed to read the informed consent aloud and we let her know that, if she gives us her consent, the session will be recorded for the subsequent interpretation of the information.

**Aspects to consider during the interview**

List of open questions to ask (if applicable):

1.- Taking medication is something that is sometimes a problem in the day-to-day life of people who have had a heart problem. What problems has taking your medication every day caused you?

2.- Since you started taking the medication, have you noticed that at certain times it is more difficult for you to take them or that you have forgotten to take the medication more often?

3.- What do you think has changed in your life since the diagnosis of the disease?

Finally, the participant is thanked for her intervention and cordially dismissed.
