# Supplementary material for: Opinions and perceptions of patients with cardiovascular disease on adherence: a qualitative study of focus groups
Source: BMC Prim Care. 2024 Feb 16;25:59. doi: 10.1186/s12875-024-02286-8 (PMC10870481; doi:10.1186/s12875-024-02286-8)
Supplement: Supplementary file 2 — Additional file 2. [file 12875_2024_2286_MOESM2_ESM.docx]

**Additional File 2**

***Guide for the moderator of the focus group***

**Aspects to consider prior to the interview**

1. Arrangement of the chairs and tables in the conference room to facilitate group dynamics and eye contact among the participants.
2. Informal conversation with attendees as they arrive.
3. Invitation for participants to help themselves to tea, coffee, or water.
4. Once all the participants are seated, provision of the patient information sheet (again) and the informed consent sheet for them to sign (in it is an explanation that the session will be video recorded and a checklist of items that the participants agree with).

**Aspects to consider at the beginning of the focus group**

1. Introduction by the group facilitators (mediator and observers) and the role of each one.
2. Introduction by the participants (name, where they live, work situation, time they are living with the disease).
3. Explanation that **a focus group is going to be carried out** (with a generic and easy-to-understand definition) and the way it is going to be carried out: an informal and relaxed group dialogue, giving space to each person’s opinion.
4. **Explanation of the rules to be followed by attendees**. Undue and/or continued interruptions and disrespect for attendees are to be avoided. Participants are reminded that they can leave the study at any time if they wish. We will make sure that there is feedback and that they understand what is going to be done, as well as the rules to follow during the course of the meeting.
5. The **purpose of the meeting (objectives)** are clarified:

The purpose of the meeting will be to understand, based on the individual testimony of each patient and the debates raised at the table, what the difficulties, barriers, and circumstances make it difficult to take their medication as prescribed. The contributions that each person makes will be used to generate a questionnaire to evaluate patients’ therapeutic adherence in the hospital/primary care environment.

**Aspects to consider during the course of the focus group**

During the course of the focus group, **non-directive open questions** related to the topic at hand may be asked, and these will be introduced as the dialogue progresses. It is important that the language be very understandable in order to gain patients’ trust. The group moderator will begin by asking this question at the beginning of the discussion:

**1.- Many people have difficulties taking their medication, how are you doing? Do you also have difficulties?**

The patients will tell personal experiences about the difficulties in routinely taking their medication.

At this point it is important to analyze the feedback from the group discussions: Who are the most involved? Have we identified patients who find it difficult to speak up? If so, recognize the causes and try to encourage participation without influencing the group dynamic too much.

Analyze non-verbal communication throughout the meeting, since this will help sense if information is being hidden or if the testimonies shared are not being truthful (task to be carried out by the observers).

If, after asking the question, we see that the patients do not have an attitude predisposed to dialogue, we can encourage participation by reinforcing **trust**: “*We are here to talk about our experiences openly and confidentially, you can feel free to express everything you want.*”

If we observe that the variable that is repeated the most is **FORGETFULNESS**:

**2. Is taking your medication a problem for your daily life?**

According to the **frequency** of forgetfulness, the following question can be posed:

**3. Can you explain some reasons that may favor forgetfulness (continuous/occasional) about taking your medication?**

If we observe that the variable that is most repeated is **SYMPTOMATOLOGY/LACK OF SYMPTOMS**

**4.- What do you do when you notice some side effect of the medication that the doctor has sent you?**

**5.- When you feel better, do you consider not taking all the medication?**

If we observe that the variable that is repeated the most is **BELIEFS:**

**6.- What do you think about the importance of adhering to the medication prescribed by your doctor?**

Other questions…

**7.- Would you know how to identify any warning signs if you did not take the medication?**

**8.- When you take the medication, do you notice improvement?**

**9.- Do you know what goals you have to have for blood pressure, weight, cholesterol levels?**

**Aspects to consider before and after the completion of the focus group**

Before the end, the moderator asks the following question:

*Before ending the meeting, do you want to add anything else that you consider relevant regarding the difficulties in taking medication?* It is a good idea to leave a margin of 10 minutes to accommodate these possible testimonials.

Finally, thank each of the patients for their participation and end the meeting. Show our availability and share our contact information in case they have any questions or suggestions.
