# Supplementary material for: Opinions and perceptions of patients with cardiovascular disease on adherence: a qualitative study of focus groups
Source: BMC Prim Care. 2024 Feb 16;25:59. doi: 10.1186/s12875-024-02286-8 (PMC10870481; doi:10.1186/s12875-024-02286-8)
Supplement: Supplementary file 3 — Additional file 3. [file 12875_2024_2286_MOESM3_ESM.docx]

**Table S1.** Representative quotations by participants. Category: lifestyle

| **Participant code** | **Quotations about lifestyle** |
| --- | --- |
| FG1P7 | *“But you lose that, and the problem with losing it is that you lose your fear and you lose the responsibility…”/ ”The source of…of adherence to medication comes from not being responsible for…for what you have. And, obviously, you have to have it from the beginning, not from the end.”* |
| FG1P8 | *“So I believe that you have to have a series of care elements, and medication is very important among them.”/ “I'm up to here with eating yogurt and fruit.”* |
| FG1P3 | *“You have to avoid fats… I think that avoiding sugar is also important because of stress, cholesterol… all of that.”/ “I try to walk as much as I can, despite having intermittent claudication that prevents me from walking every day. When I was not so limited, I walked almost 12 km a day.”* |
| FG1P1* | *“Well, I'm his wife, and like he said, he stopped smoking, so that’s good. He walks almost every morning and afternoon, and he's doing quite well.”* |
| FG1P5 | *It's like a little beer…well, okay …or if it’s a holiday and you drink two…well, okay…* |
| FG1P4* | *“From the office to the house, from the house to the office, from eating on the sofa, from the sofa to the bed, that’s what it’s like all the time. Then his legs ached, and I told him…you’re playing against the odds …”* |
| FG2P2 | *“Because I enjoy eating…So I also cook a lot and…well…I love to eat. It's just that I stuff myself and I love it…then I have a very bad time”* |
| FG2P3 | *“I was a smoker, I'm still a smoker, a little less... now I smoke half [a pack]”* |
| SIIP1 | *“I play tennis every day… I go swimming happily when I can, carry my grandchildren…and… maybe I even enjoy it a little more, right? and this... and, and... you are also a little more conscious of your health, that is... you try... I mean when the ham is right there maybe you eat a little less...”* |

FG1P7: Focus group 1, participant 7, and so on. SIIP1: Semistructured individual interview, participant 1.

* Companion of a participant with cardiovascular disease (ischemic cardiopathy).

**Table S2.** Representative quotations by participants. Category: personal beliefs

| **Participant code** | **Quotations about personal beliefs** |
| --- | --- |
| FG1P7 | *“Usually you have a heart attack and you get scared, because everyone gets scared, that's what I always say, look, I live on the second floor with no elevator, and I told my wife, I think you're going to have to take the food down here because I’m scared to go up.”* |
| FG1P6 | *“Nothing, nothing ... because if not, you live with anxiety, and that’s worse than a heart attack.”/ “What you have to do is… you have been clearly told to take medication, it’s not that complicated, and then they will have told you more or less to follow a lifestyle, you have to go here, for example, not do this, not do that... and that's it... well, try to do that and live.”* |
| FG1P3 | *“The heart endures, I held my mother who died in my arms, I could bear what happened to my son... you can’t hide things from me because... that is, the fact that you’ve had a heart attack doesn’t mean you’re useless. I mean, I have a completely normal life, I do what anyone does. What I don't do is what I shouldn't do.”* |
| FG2P1 | *“I don't know... I think it can help... because if the one to prescribe it to you is the cardiologist, who’s the one who knows your history and everything, it’ll be for a reason... I'm afraid to stop taking them... because I think they do help me...”* |
| FG2P2 | *“Sometimes I have doubts and um... it's from... I don't know, if I didn't take it I think that... tssss... Well, maybe so, and it looks like the medication isn't doing anything for me, I think so too...”* |
| FG2P3 | *“I believe that medication is sacred. Sacred, that’s what I think.”* |
| SSIP1 | *But then, was that quality of life? I mean, what are you going to be able to do? Why did they save your life? To continue living? Or to lock you up, or to be finished? You have to try to stretch it, right? everything possible…”* |

FG1P7: Focus group 1, participant 7, and so on. SIIP1: Semistructured individual interview, participant 1.

.

**Table S3. R**epresentative quotations by participants. Category: fear

| **Participant code** | **Quotations about fear** |
| --- | --- |
| FG1P8 | *“Yeah. And I took the medication… and the first month or so, well, they had to give me diazepam, and I'm taking diazepam because I couldn't sleep, I was afraid of going to sleep because I thought I wasn't going to wake up.” “The thing is I got very scared, and more or less I still am.” “What I don't want is for it to be taken away from me... because...if not, they’d have to start the routine of that again...”* |
| FG1P3 | *“My problem is coronary and obviously I am very afraid.”* |
| FG1P7 | *“This man stopped being afraid and started smoking again, the other is still afraid. So, I think the problem is that when you only have a heart attack, or angina, you overcome the fear very quickly. So, with the fear that passes very quickly, the feeling of responsibility for taking medication, for taking care of yourself, also passes. So, the problem for me is that it’s important to make people understand that when you have a heart attack, it’s not a coincidence; you have to take care of yourself.”/ “No, it's not that it's laziness, it's that the relationship you have with the disease at the beginning is fear.”/ “The problem I'm telling you is that, when you lose your fear, the conditions... the conditioning factors you have now to take the medication, you lose them.”/ “ When you are younger and you have a heart attack, at 32 years old, the fear you have is great as hell, and when the fear passes the problem is that normally…I say normally because there will be people who don't, but, normally, people go back to living life as they lived before...”* |
| FG1P6 | *“It’s fear! It is also fear of all that. By saying, well… well, let's see… The doctor has changed them [pills]* *for ones that cost me a little less. But…what do you say…it's not fear because everything is…a snowball that is getting…” “Nothing, nothing ... because if not, you live with anxiety, and that’s worse than a heart attack.”* |
| FG2P3 | *“And I'm telling you, fighting cancer, last year again, so, I say well, when it's time... just like I think I'm not going to die of a heart attack, I always tell him eh... when I go to see him...”/ “What if I get one and I die? Well, you have to die of something, what are you going to do about it?”* |
| FG2P2 | *“The information that they give you with the pills is not clear to me, because I don't want to continue reading either because you continue reading and it tells you that there is a contraindication … this, that, the other... and I say oh, I'm going to die taking them...”/ “Look, I'm going to die, but when it's my turn and when I say... because we're here and we were born, and we don't know when... and I'm going to...”* |

FG1P7: Focus group 1, participant 7, and so on. SIIP1: Semistructured individual interview, participant 1.

* Companion of a participant with cardiovascular disease (ischemic cardiopathy).

**Table S4.** Representative quotations by participants. Category: disease symptomology

| **Participant code** | **Quotations about disease symptomology** |
| --- | --- |
| FG1P8 | *“I got up at 7 in the morning to go to work on the construction site, and when I arrived at the site at 8 o'clock I began to feel bad... I had, like, some chills... a little vomiting, nausea... I held on like this until 6 in the evening when the day ended and… and I got home and told my wife.”* |
| FG1P7 | *“I had my first heart attack, before the heart attack, I started to sweat and I started to feel a little dizzy, to feel weak...”* |
| FG1P6 | *“The thing is that the first symptoms were strange, I started to sweat and such and then it went away, and I was going to go to bed, but I wasn't feeling well, I thought it was indigestion.” / “Well, I always imagined that a heart attack … well, I don't know, that you’d have chest pain, something... I don't know really, I didn't think that because I was sweating ...”* |
| FG1P5 | *“Yes, and the joints …”* |
| FG1P3 | *“I get a little tired and I stop, before I get fatigued, before I get fatigued! And if I don't, I feel... And if I don't stop, I don't know, well I think I must be a rare case because when I'm going to get tired, I notice it beforehand, when I'm walking, I say: okay, we have to stop, and I stop” / “Besides, not having symptoms, it’s like it makes me feel good.”* |
| FG1P2 | *“We lose our fear because it doesn't hurt”* |
| FG1P4* | *“That is the reality, that is the truth. Of course nothing hurts, nothing hurts... and look, until something happens” / I believe that... in the end you have to feel pain to take care of yourself, without symptoms.../ “Since nothing hurts... mmm... let's live.”/ “I I am in favor of thinking that you have to be mentalized… I mean when I get angry and say you haven't figured it out yet… I mean…if I had a little bit of pain…but since I don't have anything…well that's it…”* |
| FG2P2 | *“…Also, I’m a bit weird… (laughs). And... I said, well, I'm going to check it out... and I noticed that I was more fatigued, you know? I... I picked up my granddaughter and such and when I picked her up, and ... and then I pushed myself... to the limit.”/ “My stomach bloats up all over, it swells up a lot, huh…I don't know if it happens to you all, but but…I have tremendous fluid retention and I say…of course …from so much medication, we are getting bloated.”* |
| FG2P1 | *“Sometimes I do get a little more tired... but it's also age.”* |
| SIIP1 | *“The truth is that I never have any discomfort at all:”* |

FG1P7: Focus group 1, participant 7, and so on. SIIP1: Semistructured individual interview, participant 1.

* Companion of a participant with cardiovascular disease (ischemic cardiopathy).

**Table S5.** Representative quotations by participants. Category: beliefs about medication

| **Participant code** | **Quotations about beliefs about medication** |
| --- | --- |
| FG1P8 | *“I think that is the main thing, you have to take the medication.”* |
| FG1P5 | *“No matter how old you are... taking care of yourself and taking your medication is the main thing...”* |
| FG1P7 | *“One of the clear things is that... one of the clear things is that you have to take your medication...”* |
| FG1P4* | *“Man, the medication... that clearly has to be...”/ “Medication is sacred.”/ “Not the medication, well... the truth is that what has been a total success is Repatha... you really notice... what a drug they prescribed him!…that is the truth.” / “That medication was a total success… I told Dr. Cordero how good it had been… some levels [of blood tests]…”/ “There are medications that are not at all similar to each other…”* |
| FG2P2 | *“No… that… that I have doubts. It's not clear to me if all the medication I take really has the effect that… that, well …” / “Above all, um… the Emconcor that I take, I don't know, that pill, for me, it's very important. I stopped taking it on my own, for a few days and... when I stopped taking them, I did notice that... that I needed them... that I don't know if, I'm telling you, if it's psychological or it's...” / “Because if I told you that I’m convinced that the medication... helps me... to be better... I have doubts... it's just that I don't... I don't know. I don't know…”* |
| FG2P3 | *“I think that medication is sacred. Sacred, that’s what I think. It has never occurred to me to think well, well, I am going to take off this one or this one…or this other one…no…it is sacred.”* |
| SIIP1 | *“But really that's the only thing, now the anticoagulant that I take is, like, lighter… it's Adiro.”/ “If they give me 15 a day, well, I'm happy because for me, the important thing is not the length of life but quality, right? And then, well, that's my attitude then.”* |

FG1P7: Focus group 1, participant 7, and so on. SIIP1: Semistructured individual interview, participant 1.

* Companion of a participant with cardiovascular disease (ischemic cardiopathy).

**Table S6.** Representative quotations by participants. Category: taking medication

| **Participant code** | **Quotations about taking medication** |
| --- | --- |
| FG1P8 | *“I, for example, have it chronically, I have to take it always. Of course... that's it... I feel good or I feel bad, I have to take it.”/ “For example, I take between 6 or 7 pills a day” / “Let me tell you, I can't have too many or forget because... I going to hold out until the end.”/ “No... not fear... you take it because you have to take it.”/ “But come on, I take them every day…”/ “I have it on it like this, and it says: breakfast” [writing gesture]/ “The thing is, is that I go to my parents' house to eat and I don't take the medication with me.”* |
| FG1P6 | *“What you have to do is... you've been told clearly to take medication, it's not that complicated.”/ “No, but what... I’ve never forgotten my medication, it's a matter of habits…”/ “No, but what... I’ve never forgotten my medication, it's a matter of habits … I mean, after eating I take the pills.”* |
| FG1P5 | *“But that happens a lot at the beginning, then you go… look, even after 31 years, well, sometimes you forget.”* |
| FG1P7 | *“In my case, I take Repatha too, I mean, I prick myself with Repatha”/ “We have to hope that this gentleman (points to patient 8) is lucky enough to have that awareness of taking the medication, to adhere to the medication. Normally you lose it the moment the fear is lost.”/ “In the end, when time passes, it makes you forget one day at midday, another day in the morning, you forget, you forget. You forget for sure.”* |
| FG1P3 | *“I keep up with my follow-ups and the medication, which I don't forget. I get up at 6:30 in the morning, and always at the same time, at 7:00 in the morning the morning shot, at noon the midday shot, the snack and the evening night, that is… I never forget/ “I don't know… I… you can see that I am a little more orderly. I do go… of course, I go to my daughter's house, and I take the pill box with me.”/ “And I almost always carry the pill box in this fanny pack… so a doctor already told me… from now on you are going to take more pills than chickpeas.”* |
| FG1P1* | *“Not him, he doesn't forget” / “No, he has his pill box with his pills and…”* |
| FG2P1 | *“…I take the pills, yes…” / “I’m good about taking my medication …I go to Dr. Cordero…” / “I was fine from the beginning, I assumed I had to take it…”* |
| FG2P2 | *“I'm fine with the medication, but, well, there are times that... that I'm a bit saturated with so many pills, right?” / “I take some off eh…on my own…I’ll tell you the truth, why lie? But well, then on the other hand I say... I have to take it and... and well, uh... I have very bad cholesterol and yes, I try not to give up the cholesterol pill, just like I do with Adiro and other things, right? But hey, I'm managing, I'm not managing it great, but I'm managing it” / “...And... there are times that, for example, with the Adiro, I say ‘uff, not today’, because I've had four [pills] in the morning, then more at midday, then at night…”* |
| FG2P3 | *“Medications… I take twenty-something pills a day because they are many things apart from the heart and I have no problem taking them, that is, in the morning there are 12, at noon there are 4, and at night there are 10, and that's it.” / I've always had high blood pressure so…just for blood pressure I took four pills…it's complicated.”* |
| SIIP1 | *“Well, no, the truth is that…look, I am very consistent and very disciplined in general in everything. So then, I take them, and I take them” / “Well, not really, look, sometimes when you go on a trip... I have a son who lives in America... so I adjust the schedules and then take them there anyway, morning and night” / “Very rarely have I forgotten to take it because maybe…man, you go out at night…and you arrive late or whatever and you forget. But really that rarely happens because above all, because my husband also takes his, so usually we remember.”* |

FG1P7: Focus group 1, participant 7, and so on. SIIP1: Semistructured individual interview, participant 1.

* Companion of a participant with cardiovascular disease (ischemic cardiopathy).

**Table S7.** Representative quotations by participants. Category: Patient-health system and health professional relationship

| **Participant code** | **Quotations about the patient-health professional relationship** |
| --- | --- |
| FG1P3 | *“They are doing a fabulous follow-up on me, and I have not had anything again, I lead a totally normal life.”* |
| FG1P7 | *“Cardiac rehabilitation... which is an activity that is done after the heart attack, inside the Hospital, it’s done by people from the hospital: psychologists, psychiatrists, doctors, nurses...”/ “What we are talking about with cardiac rehabilitation... is a topic that is an investment for health... which has a cost.”/ “I believe that psychological help, psychological support and physical help in terms of ... in terms of controlling our body and knowing what our limits are after the heart attack, I think it is paramount.”* |
| FG1P8 | *“The social worker gives you some papers and she gives you an appointment for two or three years from now to…to take one of that.”/ “Of course, if you had never been to the doctor either…”* |
| FG2P1 | *“Yes of course, they explained to me what each one was for…”* |
| FG2P2 | *“I haven't asked them, but I haven't asked them because I think they don't have time to explain to me...”/ “Well, about the doctor, well, I've told him many times we talk... when I talk to him... but of course once a year... I have a check-up ... look, I come so that you can explain everything I'm taking ... each pill I take, what it’s for, what effects... but I haven't done it... the truth is...”/ “Yes, yes, but I don't have any problem with him... the doctor also seems fantastic to me.”/ “ I started at the Hospital here and each time I saw a different doctor who was…students…which I think is great because they have…I was very lucky that they took me once…which was when the angina hit me for the second time ... I just don't remember...a girl who just joined here in the consult ... who finished her degree and such and was so extraordinary...so extraordinary...with a huge interest”.* |
| FG2P1 | *“The doctor, whatever you ask her... she will resolve it for you, the doubt.”* |
| SIIP1 | *“I am extremely grateful for my life, to the Hospital de San Juan and to the ambulance and to the doctors who were with me on the tennis court who gave me [CPR]… and then an anesthesiologist came by who intubated me… or if you want to know about luck, that was mine.”* |
| FG1P7 | *“I think that one of the problems is that the medical reports that are here are not seen in Hospital La Fe, or in Madrid, or in the Basque Country... it should be information that can be shared without having to take someone's file and take it somewhere else.”* |
| FG1P6 | *“Here in the Valencian Community it hasn't been that long either because not long ago, the Hospital de San Juan couldn't see the reports from the General Hospital.”* |
| FG1P3 | *“It takes time, but ideally it should be at the national level. That you enter the [patient ID] and everything appears…”* |

FG1P7: Focus group 1, participant 7, and so on. SIIP1: Semistructured individual interview, participant 1.

**Figure 1**. Flow chart for data analysis process

**Figure 2**. Coding scheme and number of coded elements per category. The thickness of the border indicates the volume of coded elements in that category.
